# Interface between mental health and the earthquake: considering humanitarian endeavor

**DOI:** 10.3389/fpubh.2024.1326407

**Published:** 2024-02-28

**Authors:** Sara Akram, Muhammad Yousuf Jat Baloch, Abdulwahed Fahad Alrefaei, Mikhlid H. Almutairi, Muhammad Idrees, Hamad Abdulla R. A. Al-Kubaisi

**Affiliations:** ^1^School of Sociology and Anthropology, Sun Yat-Sen University, Guangzhou, China; ^2^College of New Energy and Environment, Jilin University, Changchun, China; ^3^Department of Zoology, College of Science, King Saud University, Riyadh, Saudi Arabia; ^4^Department of Rural Sociology, University of Agriculture Faisalabad, Faisalabad, Pakistan; ^5^Qatar Investment Authority, Doha, Qatar

**Keywords:** psychological sickness, Afghanistan, earthquake, humanity survival, coping mechanism

## Background

The state of one's mental wellbeing is an essential aspect of one's total health, playing a pivotal role in personal, communal, and economic development. Exposure to unfavorable socioeconomic, political, and ecological circumstances, such as injustice, inequalities, impoverishment and catastrophes resulting from nature, increased one's chance of experiencing psychological disorders (1). Throughout a span of 40 years, the citizens of Afghanistan have endured a tumultuous era marked by internal conflicts, an unsteady economy, and frequent natural calamities, resulting in a significant prevalence of mental illnesses. Approximately 47% of the population experiences mental health problems (2). Moreover, the negative perception around mental health acts as a barrier to obtaining easily accessible mental healthcare, particularly in rural regions, population characterized by limited educational opportunities (3). An earthquake with a magnitude of 6.3 occurred on October 7, 2023, devastating the communities and destroying dozens of clay-brick houses that were not strong enough to endure the first shock and its subsequent aftershocks. Schools, hospitals, and other community institutions were also ruined (4). According to government officials, ~2,000 people have been killed in Herat province, with more than 90% of those killed being women and children (5). This catastrophe further weakened the country's mental health. Furthermore, the restricted availability of mental health services has presented difficulties in addressing the psychological consequences of traumatic occurrences such as earthquakes. In August 2021, following the Taliban's seizure of power, the foreign fund stopped providing coverage for 79.3% of health costs (6, 7). Mental health concerns do not solely manifest among the Afghan population during times of war-induced violence. The aforementioned challenges arise from everyday pressures linked to human insecurity. These pressures consist of the repercussions of housing insecurity and lack of means of subsistence, forced migration, the disruption of familial and communal ties, the resultant destitution and catastrophes like earthquake (8). The intersection of societal stigma and socioeconomic inequality has resulted in a multifaceted approach to providing assistance to individuals with emotional or behavioral disorders.

Even though there is literature available on the mental illness health status in Afghanistan before the COVID-19 outbreak, there is a significant lack of information regarding the years following the pandemic, particularly in relation to the recent earthquake of 2023. Therefore, this study investigates the impact of the ongoing earthquake occurrence on the mental health of individuals in Afghanistan and suggests strategies to address this problem.

The study employed secondary data to enhance the coherence and reliability of its reasoning. The research sources utilized for the current study included Google, Google Scholar, Microsoft Academic, JSTOR, and Academia.edu. Using keyword searches such as “Afghanistan earthquake, mental health, humanity sufferings, immediate rescue, country's health system, mental health priorities available facilities, NGOs response to tragedy and coping mechanism,” the study attempts to identify its desired purpose.

## The consequences of the earthquake on the psychological state

Earthquakes intensify the occurrence of psychiatric health illnesses, including depression, anxiety, post-traumatic stress disorder (PTSD), insomnia issues, and drug dependence (2, 9). The psychological reaction to a catastrophe can be evaluated through five separate phases: alert issued effects, inspection, immediate recuperation, and prolonged recuperation. When an earthquake occurs, the reaction usually starts at the impact stage and might result in intense feelings (10). Afghanistan, situated within the rugged “Hindu Kush” Himalayan range, is recognized as the second most seismically active area in the world. Moreover, the collision of the Indian and Eurasian tectonic plates at the border of Afghanistan and Pakistan has made the country vulnerable to seismic events and the leading to psychosocial side effects (11).

The frequency of psychological disorders in war-affected areas is more than twice as high as in the overall population, as demonstrated by data from the World Health Organization. The prevailing rate of mental health difficulties among the adult population is 22%, with 17% having mild to moderate depression, anxiety, and post-traumatic stress disorder (PTSD), while 5% encountering severe depressive symptoms, stress, PTSD, schizophrenia, and or bipolar disorder (12). Another study conducted in Afghanistan in 2021 by BMC Psychiatry investigated eight different areas and revealed concerning prevalence rates of depression and anxiety disorders among the whole population. Around 47.12% of the surveyed respondents reported experiencing a notable level of discomfort in the month prior, whilst more than 39.44% stated that their mental health problems had adversely affected their everyday functioning (13).

The recent earthquake in western Afghanistan had a profound impact on the countryside human population, resulting in substantial health concerns, particularly for psychological issues (14). Factors contributing to these risks included physical trauma, bereavement, housing loss, financial hardship, the displacement, female suffering, loss of relatives and low educational attainment (15–17). However, mental health remains a highly stigmatized subject in Afghanistan, despite the shared experiences of many individuals, mostly owing to a complex combination of cultural, religious, economical, and environmental variables. Currently, the mental health sector has been given lower priority by the de-facto Taliban administration due to the deteriorating economy and high levels of poverty, resulting in it being mostly neglected (13). The lack of local mental healthcare, geographical obstacles and inadequate transportation infrastructure impede prompt response efforts and heighten the likelihood of enduring societal consequences. These consequences may manifest as societal dysfunction, resulting in escalated family disputes, violence, aggressiveness, suicidal ideation, and drug misuse.

## Obstacles and ramifications

The country's mental healthcare administration is currently facing multiple issues, which have been further exacerbated by the devastating earthquake. Poverty stands as the foremost concern, as evidenced by the latest International Wealth Index data indicating that a significant 98% of families residing in Herat Province endure life in poverty (18). The earthquake inflicted significant destruction to many dwellings, leading to the evacuation of residents that lack the financial means to reconstruct. Moreover, it is quite probable that the earthquake contributed to the onset of emotions such as sadness, unease, post-traumatic stress disorder, and severe depressive illness (19–22). If the region did not suffer from low accessibility and a scarcity of psychological healthcare facilities, psychiatric health practitioners would be able to effectively address these psychological problems (22, 23). Herat's asphalt roads are insufficient, forcing the populace to utilize dirt tracks that wind across steep terrain. Because of the earthquake's surface rupture and ground displacement, these dirt roads are now worthless, which delays access to medical services (24, 25). A lot of Muslims regard the calamity as a trial, or test from God, which might psychologically deter individuals from seeking mental health care. Earthquakes can also reinforce society's fatalistic attitudes (26, 27). Numerous new societal problems are a result of people's lack of awareness of the possible growth in mental diseases. Because of the long-running conflict and the country's poor mental health, Afghanistan has seen massive drug usage (28, 29). Research has shown a reciprocal relationship between persons with PTSD or MDD and drug addiction, which may make the problem worse by encouraging more people to use drugs (30). In addition, a research published in 2021 found that MDD patients had violent outbursts far more frequently (21, 31). This could cause family conflicts in Afghanistan, where mixed-family families make up the majority of the population (20, 31). The continuation of a detrimental cycle, in which the limited accessibility of psychological healthcare for individuals results in substance abuse and aggression, worsening the overall situation and putting additional pressure on already overwhelmed mental care facilities, emphasizes the urgent need for the government and supporting non-governmental organizations to take proactive measures in addressing mental health interventions from the start and in the future.

## Initiatives aimed and suggestions

Various organizations have addressed the pressing demand for mental health centers in Afghanistan. Health-Net TPO, a Dutch organization established by Médecins Sans Frontières, has been delivering treatments in Afghanistan ever since 1994. Since 2005, the company has solely concentrated on integrating mental health services into healthcare institutions in Afghanistan (32).

The training program offered by Health-Net TPO has facilitated the acquisition of essential skills and information for 325 persons from Afghanistan, enabling them to operate as proficient psychosocial specialists. These experts are now capable of providing assistance to humanitarian citizens at medical services. Furthermore, the organization has provided comprehensive training to more than 250 healthcare practitioners, encompassing doctors, obstetricians and nursing staff in the field of psychological disorders health. Furthermore, they have successfully created 56 mental health facilities across the country (6).

Health-Net TPO teams are now offering aid to individuals in the Herat and Farah provinces, which have been affected by the crisis. Mobile healthcare teams have been dispatched around the region to provide essential emergency medical treatment and offer psychological assistance to those affected (33). A group of six psychosocial therapists has begun providing psychological first aid and mental health support to children and their families who have experienced considerable psychosocial distress. A combined total of 1,600 dignity kits (DKs) are sent to the districts of Herat and Farah. This distribution is part of a joint effort with the United Nations Population Fund (UNFPA) and Health-Net TPO (33).

While these groups provide essential emergency aid, the availability of mental health counseling is inadequate. Through cooperation with various international organizations, the incorporation of religion into mental health practices would reduce societal stigma associated with psychological therapy and promote individuals' willingness to seek expert guidance. Global restrictions on emergency assistance, including medical and psychiatric aid, are soon to be lifted to alleviate the growing burden on mental health. By allowing the influx of help, this will simultaneously allow those in need to obtain mental healthcare in adjacent countries that have better facilities (34).

Evidence has indicated that mental healthcare services effectively mitigate the likelihood of chronic illnesses associated with insufficient counseling, such as panic attacks, anxiousness, and drug dependence (35–37). Therefore, it is crucial for global leaders, together with non-governmental organizations, to adopt a more assertive approach in providing psychological heath solutions in Afghanistan. Considering the current situation in Afghanistan, where people are experiencing considerable psychological anguish, it is of utmost importance to prioritize the resolution of this matter, given that half of the population is impacted by psychological stress (38).

In order to mitigate the effects of extensively impaired infrastructure, a practical approach is to enhance the use of telemedicine in collaboration with Afghan Cellular, the leading telecoms supplier in Afghanistan. By implementing this, it would allow individuals in rural regions to obtain mental health assistance, while also providing the benefit of limited identification. Technological elements such as speech filters enhance communication by allowing individuals to hide their identities. The dominant conservative ideologies in rural Afghanistan impede women's ability to obtain counseling services. In order to tackle this problem, it would be advantageous to establish counseling teams that have a balanced gender composition. This would allow women to engage with female counselors in a manner that is seen socially acceptable by the local community. Camps aimed at aiding victims should incorporate recreational activities to effectively channel the energy of the young individuals, a strategy that has been proven to improve their mental wellbeing (39, 40). Employment not only generates financial income but also improves the mental wellbeing of numerous individuals, especially women. It is recommended to incorporate activities such as sewing or tailoring within a holistic rehabilitation approach (41). The effectiveness of such activities depends on engaging in transparent and sincere communication with government officials and significant religious figures. Their support for such organizations has the potential to greatly increase the Afghan community's willingness to seek mental health assistance.

Since the Taliban took control of the country in August 2021, major political shifts and devastating natural disasters have made Afghanistan's situation more difficult. The predominance of women and children among the numerous casualties resulting from the earthquakes in the province of Herat underscores the inherent weaknesses of Afghan dwellings constructed with sun-dried bricks. In addition, the Taliban's restriction on women's access to non-governmental organizations (NGOs) has hindered the delivery of vital health and humanitarian services. The issue is exacerbated by international resolutions to decrease financial assistance. The convergence of crises has had a disproportionate impact on women and children, as it has led to possible surges in infections, depression, anxiety, insecurity and starvation (42).

Sanctions and decreases in assistance are used as measures to enforce accountability on the Taliban, but unintentionally inflict harm on women and children in Afghanistan. This scenario strongly implores the entire community to take action and avert any natural or political catastrophes that may pose a threat to the most susceptible individuals in society. It is essential to differentiate between humanitarian assistance and political matters, actively participate in productive dialogue with the Taliban to find practical solutions, and uphold the key role of non-governmental organizations, especially considering the frequency of natural calamities. The current scenario underscores the utmost importance of the international community giving priority to humanitarian concerns rather than political disputes. This is essential to ensure that aid reaches the most susceptible populations and that non-governmental organizations (NGOs) can continue their operations despite persistent societal challenges (42).

## Conclusion

Afghans have had persistent psychological health difficulties for numerous years; nonetheless, the recent earthquake has heightened concerns around psychological wellness. Responding to mental health needs in this region is difficult due to its distant location, the ongoing humanitarian situation, and the societal stigma surrounding mental health. International organizations should allocate greater resources toward mental health services and ensure sufficient training of healthcare professionals to effectively cater to the mental health requirements of the Afghan population, employing innovative initiatives and rehabilitation methodologies. Furthermore, it is imperative to enhance public consciousness regarding mental health in order to eradicate the associated social stigma.

## Author contributions

SA: Conceptualization, Formal analysis, Writing – original draft, Data curation, Investigation, Methodology, Project administration, Software, Validation, Writing – review & editing. MB: Conceptualization, Writing – original draft, Writing – review & editing, Formal analysis, Investigation, Methodology, Project administration, Resources, Software, Supervision. AA: Conceptualization, Formal analysis, Resources, Writing – original draft, Writing – review & editing, Data curation, Funding acquisition, Investigation, Project administration, Supervision, Validation, Visualization. MA: Project administration, Supervision, Validation, Writing – original draft, Writing – review & editing, Conceptualization, Funding acquisition, Investigation, Methodology, Resources, Visualization. MI: Conceptualization, Project administration, Writing – original draft, Writing – review & editing, Data curation, Formal analysis, Investigation. HA-K: Writing – review & editing, Funding acquisition.

## References

[1] WHO. Addressing Mental Health Through Primary Care and Community Engagement in the WHO South-East Asia Region. Geneva: WHO. (2022).

[2] Kovess-Masfety V Keyes K Karam E Sabawoon A Sarwari BA. A national survey on depressive and anxiety disorders in Afghanistan: a highly traumatized population. BMC Psychiat. (2021) 21:1–12. 10.1186/s12888-021-03273-4 34158003 PMC8218387

[3] Nine SB Najm AF Allan EB Gronholm PC. Mental health stigma among community members in Afghanistan: a cross-sectional survey. Int J Social Psychiat. (2022) 68:1470–85. 10.1177/00207640211036169 34369169

[4] NBC News. Afghanistan is Hit by Fourth 6.3-Magnitude Quake in Just Over a Week. New York, NY: NBC News. (2023).

[5] Coren A Chen H. We Are Left With Nothing': Survivors of Afghanistan Earthquake Tell of Trauma and Heartbreak (2023). Retrieved from: https://edition.cnn.com/2023/10/14/world/afghanistan-earthquake-herat-survivors-dst-intl-hnk/index.html

[6] Hussaini SJ Ali SH Rahmat ZS Islam Z Tharwani ZH. Mental health impacts of earthquake on Afghans amidst humanitarian crisis. Ann Med Surg. (2022) 81:104521. 10.1016/j.amsu.2022.104521 36091197 PMC9460552

[7] Essar MY Ashworth H Nemat A. Addressing the humanitarian crisis in Afghanistan through $10 billion Afghani assets: what are the challenges and opportunities at hand? Global Health. (2022) 18:1–4. 10.1186/s12992-022-00868-8 35907893 PMC9338494

[8] Alemi Q Panter-Brick C Oriya S Ahmady M Alimi AQ Faiz H. Afghan mental health and psychosocial well-being: thematic review of four decades of research and interventions. BJPsych open. (2023) 9:e125. 10.1192/bjo.2023.502 37424447 PMC10375890

[9] Pollice R Bianchini V Marola V Verni L Di Mauro S Ussorio D. Post-traumatic and psychiatric symptoms among young earthquake survivors in primary care camp hospital. Eur J Inflammat/. (2011) 9:39–44. 10.1177/1721727X1100900106

[10] Morganstein JC Ursano RJ. Ecological disasters and mental health: causes, consequences, and interventions. Front Psychiatry. (2020) 11:1. 10.3389/fpsyt.2020.00001 32116830 PMC7026686

[11] Rehman K Burton PW. Seismicity and seismic hazard parameters in and around Pakistan. J Seismo. (2020) 24:635–53. 10.1007/s10950-020-09917-4

[12] Noh J-W Lee LJ Kim K-B Cha J Kwon YD. Factors influencing injury or death due to traumatic events in Afghanistan's crisis-affected populations: a cross-sectional nationwide study. BMJ Open. (2022) 12:e063329. 10.1136/bmjopen-2022-063329 36576193 PMC9723898

[13] Schwartz L Lane H Hassanpoor Z. Overview and understanding of mental health and psychosocial support in Afghanistan. Int Health. (2023) 15:601–7. 10.1093/inthealth/ihad055 37490026 PMC10472885

[14] The New Humanitarian. A Week of Earthquakes Brings Death, Grief, and Trauma to Afghanistan's Herat. Geneva: The New Humanitarian. (2023).

[15] Nakajima S Masaya I Akemi S Takako K. Complicated grief in those bereaved by violent death: the effects of post-traumatic stress disorder on complicated grief. Dialogues Clin Neurosci. (2022) 14:210–4. 10.31887/DCNS.2012.14.2/snakajima 22754294 PMC3384450

[16] UN News. 500 still missing following Afghanistan earthquake, say UN aid teams. UN News. Retrieved from: https://news.un.org/en/story/2023/10/1142107

[17] WHO. Most Casualties in Recent Afghan Earthquakes Are Women, Children -WHO (2023). Retrieved from: https://www.reuters.com/world/asia-pacific/most-casualties-recent-afghan-earthquakes-are-women-children-who-2023-10-09/

[18] Lab GD. GDL Area Profile Report West (Ghor Herat Badghis Farah) (Afghanistan) (2023). Retrieved from: https://globaldatalab.org/areadata/profiles/AFGr108/

[19] Vos SR Clark-Ginsberg A Puente-Duran S Salas-Wright CP Duque MC Herrera IC. The family crisis migration stress framework: a framework to understand the mental health effects of crisis migration on children and families caused by disasters. New Dir Child Adolesc Dev. (2021) 2021:41–59. 10.1002/cad.20397 33634569

[20] Catani C Schauer E Neuner F. Beyond individual war trauma: domestic violence against children in Afghanistan and Sri Lanka. J Marital Fam Ther. (2008) 34:165–76. 10.1111/j.1752-0606.2008.00062.x 18412824

[21] Liu Q Cole DA. Aggressive outbursts among adults with major depressive disorder: results from the Collaborative Psychiatric Epidemiological Surveys. J Psychiatr Res. (2021) 135:325–31. 10.1016/j.jpsychires.2021.01.040 33556687

[22] Zhang J Li G Yang H Cao C Fang R Liu P. The main effect and gene-environment interaction effect of the ADCYAP1R1 polymorphism rs2267735 on the course of posttraumatic stress disorder symptoms—a longitudinal analysis. Front Psychiatry. (2022) 13:1032837. 10.3389/fpsyt.2022.1032837 36386994 PMC9650374

[23] Ahmad DA. Mental Health in Afghanistan - When the Mind Suffers (2017). Retrieved from: https://tolonews.com/opinion/op-ed/mental-health-afghanistan-when-mind-suffers

[24] News. Afghanistan is hit by fourth 6.3-magnitude quake in just over a week. NBC News (2023). Retrieved from: https://www.nbcnews.com/news/world/afghanistan-fourth-earthquake-herat-rcna120541

[25] web R. Afghans Face Deep Trauma Following Third Powerful Earthquake. (2023). Available online at: https://reliefweb.int/report/afghanistan/afghans-face-deep-trauma-following-third-powerful-earthquake

[26] Aksa FI. Islamic perspectives in disaster: An alternative to changing fatalistic attitudes. Jàmbá. (2020) 12:1. 10.4102/jamba.v12i1.942 33354304 PMC7736663

[27] Fahm AO. Islam and disaster management in contemporary times: a psycho-socio-spiritual response. J Religion Spirituality Social Work. (2019) 38:259–80. 10.1080/15426432.2019.1632246

[28] UNODC. Drug use in Afganistan 2009 (2009). Retrieved from: https://www.unodc.org/documents/data-and-analysis/Studies/Afghan-Drug-Survey-2009-Executive-Summary-web.pdf

[29] Shalizi H. Traumatized Afghans Addicted to Heroin (2007). Retrieved from: https://www.reuters.com/article/lifestyle-afghan-drugs-dc/traumatized-afghans-addicted-to-heroin-idUKISL2762520071016

[30] Simmons S Suárez L. Substance abuse and trauma. Child Adolesc Psychiat Clini. (2016) 25:723–34. 10.1016/j.chc.2016.05.006 27613348

[31] Bjork JM Grant SJ. Does traumatic brain injury increase risk for substance abuse? J Neurotrauma. (2009) 26:1077–82. 10.1089/neu.2008.0849 19203230 PMC2989860

[32] Hilhorst D Pereboom E. Dutch Humanitarian Aid: now and in the future. A sector consultation in preparation of the Netherlands Humanitarian Summit 2015. In: Humanitarian Aid and Reconstruction. Wageningen: Wageningen University/Globalisation (2014).

[33] HealthNetTPO. Afghanistan Earthquake: HealthNet TPO Emergency Response (2023). Retrieved from: https://reliefweb.int/report/afghanistan/afghanistan-earthquake-healthnet-tpo-emergency-response#:~:text=Our%20response,first%20aid%20to%20those%20affected

[34] WHO. Who-Led Health Cluster Appeals for $7.9 Million To Provide Health Services to 114,000 People Most Affected By Earthquakes In Western Afghanistan. (2023).

[35] Alemi Q Stempel C Koga PM Montgomery S Smith V Sandhu G. Risk and protective factors associated with the mental health of young adults in Kabul, Afghanistan. BMC Psychiatry. (2018) 18:1–10. 10.1186/s12888-018-1648-4 29562881 PMC5863364

[36] Panter-Brick C Eggerman M Mojadidi A Mcdade TW. Original Research Article Social Stressors, Mental Health, and Physiological Stress in an Urban Elite of Young Afghans in Kabul. (2008). 18663740 10.1002/ajhb.20797

[37] Catani C Schauer E Elbert T Missmahl I Bette JP Neuner F. War trauma, child labor, and family violence: Life adversities and PTSD in a sample of school children in Kabul. J Traum Stress. (2009) 22:163–71. 10.1002/jts.20415 19462436

[38] Saleem SM Shoib S Dazhamyar AR Chandradasa M. Afghanistan: decades of collective trauma, ongoing humanitarian crises, Taliban rulers, and mental health of the displaced population. Asian J Psychiatr. (2021) 65:102854. 10.1016/j.ajp.2021.102854 34537535

[39] Sayed GD. Mental health in Afghanistan: Burden, Challenges and the Way Forward (2011). Retrieved from: https://documents.worldbank.org/en/publication/documents-reports/documentdetail/692201467992810759/mental-health-in-afghanistan-burden-challenges-and-the-way-forward

[40] Eggerman M Panter-Brick C. Suffering, hope, and entrapment: Resilience and cultural values in Afghanistan. Soc Sci Med. (2010) 71:71–83. 10.1016/j.socscimed.2010.03.023 20452111 PMC3125115

[41] Virgolino A Costa J Santos O Pereira ME Antunes R Ambrósio S. Lost in transition: a systematic review of the association between unemployment and mental health. J Mental Health. (2022) 31:432–44. 10.1080/09638237.2021.2022615 34983292

[42] Ramozi M Kaneda Y Barati H Ozaki A Mousavi SH. Earthquakes and Taliban Decrees: The Plight of Afghan Women and Children. Cureus. (2023) 15:11. 10.7759/cureus.48663 38090400 PMC10713278

